# Vascular degeneration and retinal remodeling in rd10 mice: correlating OCT, OCTA, and histological findings

**DOI:** 10.3389/fnana.2025.1683877

**Published:** 2025-10-21

**Authors:** Henar Albertos-Arranz, Xavier Sánchez-Sáez, Oksana Kutsyr, Laura Fernández-Sánchez, Carla Sánchez-Castillo, Pedro Lax, Nicolás Cuenca

**Affiliations:** ^1^Department of Physiology, Genetics and Microbiology, University of Alicante, Alicante, Spain; ^2^Department of Optics, Pharmacology and Anatomy, University of Alicante, Alicante, Spain; ^3^Alicante Institute for Health and Biomedical Research (ISABIAL), Alicante, Spain; ^4^Ramón Margalef Institute, University of Alicante, Alicante, Spain

**Keywords:** retinal remodeling, vascular degeneration, OCT, OCTA, retinitis pigmentosa, Müller cells, rd10

## Abstract

**Introduction:**

Identifying long-term changes in retinal structure and vasculature is essential for interpreting *in vivo* imaging techniques such as optical coherence tomography (OCT) and OCT angiography (OCTA) in retinal diseases. We characterized long-term alterations in vasculature, retinal structure, and glial cells by combining immunohistochemistry (IHC) with OCT and OCTA in a murine model of retinitis pigmentosa.

**Methods:**

Transversal retinal sections and wholemount retinas from C57BL/6J and rd10 mice, aged P20 to 24 months, were immunostained to evaluate retinal structure, glial cells, retinal pigment epithelium (RPE), and the vascular network. OCT and OCTA images from the central retina were also analyzed.

**Results:**

Significant retinal remodeling in the inner retina occurs over time and was detectable from 4 months using IHC and from 6 months using OCT. Remodeling was characterized by glial activation (reactive gliosis) and the formation of hyperreflective columns, which contain Müller cells, activated microglia, RPE, and choroidal vessels in the late stages. No significant differences were observed between OCTA and IHC vascular density of the superficial vascular plexus (SVP) and deep capillary plexus (DCP) in rd10 mice at any time points, except at 2 months (SVP, *p* = 0.009; DCP, *p* = 0.001). This seems a critical stage, suggesting differing rates of blood flow reduction and structural vessel loss. A peak of vascular degeneration in the SVP of rd10 mice was detected by OCTA between 2 and 6 months (*p* = 0.003), but not by IHC. Vascular degeneration peak of DCP in rd10 was observed between P20 and 2 months using OCTA (*p* < 0.0001), and between 2 and 6 months using IHC (*p* = 0.003).

**Conclusion:**

Overall, OCTA and IHC yielded comparable long-term vascular density results, supporting OCTA as a reliable, non-invasive tool for studying vessel degeneration in animal models. Therefore, longitudinal *in vivo* evaluation of retinal remodeling through OCT and OCTA constitutes a valuable methodology for investigating disease mechanisms and guiding therapeutic development.

## 1 Introduction

Retinitis pigmentosa (RP) is a genetically heterogeneous disorder defined by a progressive retinal degeneration that starts with the death of rod photoreceptors which may lead to complete blindness. Different animal models of RP have been studied, one of them being the rd10 mouse: a model of autosomal recessive RP which presents a spontaneous mutation in the Pde6b gene (Chang et al., 2002). Compared to other RP models, the rd10 mouse presents a retinal degeneration rate suitable to be considered as a good model for therapy and for the study of the RP progression. Indeed, this model is one of the most widely used for testing retinal neuroprotection (Campello et al., 2020; Valdés-Sánchez et al., 2019; Roche et al., 2016b,^2019^).

Different comparative studies have been done describing the rd10 model degeneration using optical coherence tomography (OCT) and immunohistochemistry (Pennesi et al., 2012; Hasegawa et al., 2016; Rösch et al., 2014; Gargini et al., 2007; Barhoum et al., 2008). However, although the rd10 mouse is a RP model widely used in vision science, there are no detailed studies of the long-term remodeling events derived from retinal and vascular degeneration, which are essential for understanding retinal cell changes in RP. According to the previously described (Cuenca et al., 2014a) classification in four remodeling phases, the last stage is the one that is characterized by a global remodeling of retinal connections and structures, due to the complete loss of photoreceptors.

Furthermore, the development of the OCT technology allowed the observation of vascular plexuses following the emergence of the OCT angiography (OCTA) imaging technique. This non-invasive technique acquires images of the retinal vessels by detecting the blood flow (Kashani et al., 2017; Spaide et al., 2018). Before the introduction of OCTA, the existing techniques, such as fluorescein angiography (Rösch et al., 2014) and indocyanine green (Pinilla et al., 2016) did not allow the observation of clear vascular degeneration *in vivo* in RP models. Nowadays, these vascular alterations can be detected *in vivo* using OCTA, providing faster and higher-resolution results (Cuenca et al., 2020).

Here, we provide new results of long-term changes in the retinal structure and glial network of rd10 mice, as well as the retinal vasculature, by combining OCT and OCTA imaging with immunohistochemistry (IHC). Moreover, we provide resources for *in vivo* identification of different events in long-term retinal remodeling, which may also be applicable to other models of retinal degeneration.

## 2 Materials and methods

### 2.1 Mice

The same number of male and female C57BL/6J and homozygous B6.CXBI-Pde6brd10/J (rd10) mice with a C57BL/6J background (The Jackson Laboratory, Bar Harbor, ME) were maintained in the animal facility of the University of Alicante under specific-pathogen-free (SPF) conditions with controlled conditions of temperature (23°C ± 1°C) and humidity (55%–60%) and with a light/dark cycle of 12:12 h. Both groups of animals were kept at 50 lux throughout the light cycle. Health status was monitored regularly by the animal facility. Mice were used on postnatal days (P) 20, P30, P40, P60 and at 4, 6, 12, and 24 months of age. All animals were anesthetized with a ketamine/xylazine mixture (ketamine 100 mg/kg and xylazine 10 mg/kg) for the procedures, and upon completion, while still under deep anesthesia, they were euthanized by cervical dislocation to minimize suffering. Death was confirmed by the absence of respiration and heartbeat.

This work was performed according to current regulations for the care and use of laboratory animals (NIH, ARVO, and European Directive 2010/63/EU) and followed the ARRIVE 2.0 guidelines to ensure transparent reporting, minimization of animal suffering and application of the reduction principle. All experiments in this research received prior approval from the ethics committee for animal care and use at the University of Alicante (UA-07/22/2013).

Sample size was determined based on previous studies using similar models and was considered sufficient to detect biologically relevant differences (Kutsyr et al., 2020). Animals were randomly assigned to experimental groups taking in account the genotype, sex and age. No animals were excluded from the analysis unless technical problems prevented acquisition of usable images (exclusion criteria are detailed in Section “2.4 Quantitative analysis of vascular plexuses”).

### 2.2 Immunohistochemistry

Histological studies of the retinas were performed following established procedures (Campello et al., 2020; Noailles et al., 2014). Vertical sections and retinal wholemounts preparations were collected for immunolabeling. Briefly, the enucleated eyes were fixed at room temperature (RT) with 4% paraformaldehyde (PFA) for 1 h for transversal sections and 15 min for the wholemount analysis. They were then washed in 0.1 M phosphate buffer pH 7.4 (PB) and cryoprotected in increasing sucrose concentrations (15%, 20% and 30% w/v sucrose). After the removal of the cornea, lens, and vitreous humor, the eyecups were either embedded in Tissue-Tek OCT (Sakura Finetek, Zoeterwouden, Netherlands) and frozen in liquid nitrogen to obtain fourteen-micrometer-thick transverse sections using a cryostat, or flat-mounted with the photoreceptor layer side up. The wholemount retinas were first incubated for 5 min in 2.28% sodium m-periodate (Sigma, St. Louis, MO, USA) in PB, followed by 5 min incubation in 0.02% sodium borohydride (Panreac, Barcelona, Spain) in PB, both at RT.

For immunostaining, retinal sections were washed in PB and incubated for 1 h in blocking solution (10% v/v donkey serum in PB with 0.5% Triton X-100). Next, sections were incubated overnight (RT) with the primary antibodies diluted in PB with 0.5% Triton X-100. To avoid any potential interference, the following pairwise combinations were used: mouse anti-CRALBP (1:500; Abcam, Cambridge, United Kingdom) was combined with rabbit anti-GFAP (1:50; Dako, Santa Clara, CA, USA), and goat anti-collagen type IV (1:1000; Chemicon-Millipore, Temecula, CA, USA) was combined with rabbit anti-Iba1 (1:1000; Wako Chemicals, Richmond, VA, USA). After, sections were washed in PB and incubated for 1 h with the secondary antibodies: anti-mouse-Alexa Fluor 488, anti-rabbit-Alexa Fluor 488, anti-rabbit-Alexa Fluor 555, anti-mouse-Alexa Fluor 555, or anti-goat-Alexa Fluor 633 (1:100, Molecular Probes, Eugene, OR, USA). Also, nuclear marker TO-PRO-3 iodide (Molecular Probes) was added at a 1:1000 dilution when indicated.

In the case of wholemounts, after blocking with 10% donkey serum in PB plus 0.5% triton X-100 for 1 h, the retinas were incubated for 3 days at 4 °C under agitation with the primary antibody goat anti-collagen type IV (1:1000; Chemicon-Millipore). Next, the retinas were washed in PB and incubated for 1 day at 4 °C with the secondary antibody donkey anti-goat-Alexa Fluor 488 (1:100, Molecular Probes).

Lastly, sections and wholemounts were washed in PB and mounted with Citifluor (Citifluor Ltd., London, United Kingdom) under a coverslip. Images were taken with a Leica TCS SP8 confocal laser-scanning microscope (Leica Microsystems, Wetzlar, Germany).

### 2.3 OCT and OCTA imaging

Spectralis OCT with the OCT angiography module (Heidelberg Engineering, Germany) was used to obtain high-resolution images of the central retina and the retinal vascular plexuses. Retinal volume scans (2.7 mm × 1.4 mm; 512 frames) were acquired in OCT/OCTA. For this procedure, mice were anesthetized through intraperitoneal injection of a ketamine/xylazine mixture (ketamine 100 mg/kg and xylazine 10 mg/kg) and depth of anesthesia was verified by the absence of the pedal withdrawal reflex. After that, the animals were kept on a heating pad at 37 °C and monitored throughout the procedure to ensure stable physiological conditions. Topical tropicamide 1% (Alcon Cusí, Barcelona, Spain) was used as mydriatic agent. Physiological saline (0.9% NaCl) was used to maintain corneal hydration during image acquisition, preventing drying and preserving tissue transparency. The contact lens further protected the cornea, preventing cataract formation and improving image quality.

### 2.4 Quantitative analysis of vascular plexuses

Manual segmentation of the superficial vascular plexus (SVP) and deep capillary plexus (DCP) was carried out to generate separate images of each plexus from both immunohistochemistry (IHC) and OCTA data. Final images were obtained using maximum intensity Z-projections. Images with poor quality or those in which artifacts could not be adequately reduced were discarded from the analysis. Subsequently, vascular density for both the SVP and DCP was quantified using AngioTool software (version 0.6a; National Cancer Institute, Bethesda, MD, USA) (Zudaire et al., 2011) from the processed IHC and OCTA images. Investigators performing quantitative analysis were blinded to genotype and age to minimize bias. Analysis was performed in mice at P20 (rd10, *n* = 9; C57BL/6J, *n* = 13), 2 months (m) (rd10, *n* = 8; C57BL/6J, *n* = 8), 6 m (rd10, *n* = 5; C57BL/6J, *n* = 8) and 12 m (rd10, *n* = 11; C57BL/6J, *n* = 10). The area of interest of all images was delimited at the superior central retina, close to the optic nerve.

### 2.5 Statistical analysis

Statistical analysis for the vascular density was performed using the IBM SPSS software package version 29.0.2.0. Descriptive statistics were performed to summarize the data. Changes in disease progression were evaluated over time for each group (C57BL/6J and rd10 mice) and each technique (IHC and OCTA). Moreover, within each group, differences between IHC and OCTA methods were analyzed. Finally, for each method, differences between C57BL/6J and rd10 mice were assessed. These analyses were conducted using the One-way or Two-way ANOVA tests with Bonferroni *post hoc* correction for multiple comparisons. Graphical representations were created using the GraphPad Prism 9.4.1 (GraphPad Software, San Diego, CA, USA) and statistical significance was set at *p* < 0.05.

## 3 Results

### 3.1 The rd10 retina undergoes a severe remodeling process once the outer nuclear layer has been completely lost

**FIGURE 1:**
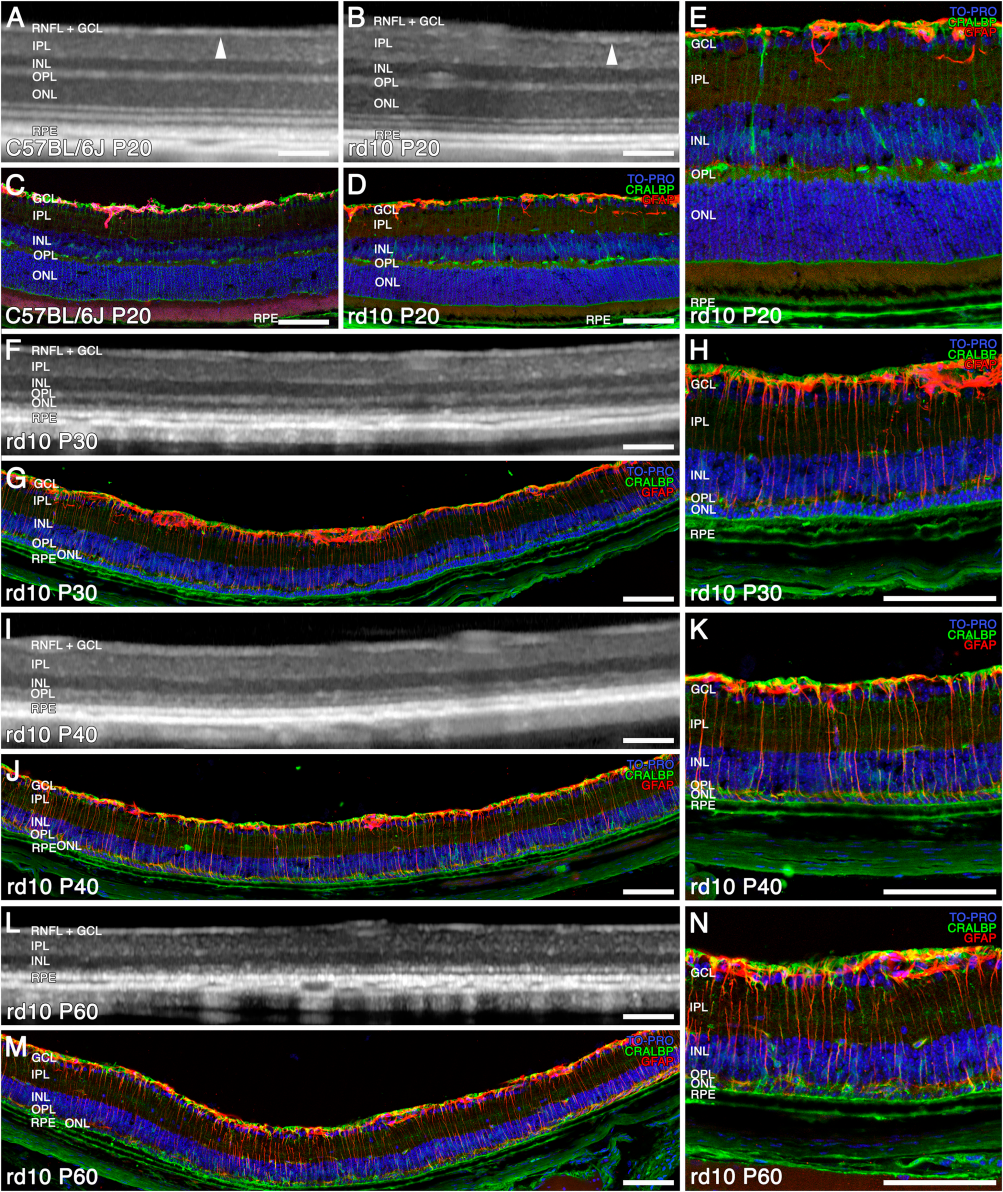
Structural and morphological changes of C57BL/6J and rd10 retinas observed with OCT **(A,B,F,I,L)** and immunohistochemistry **(C–E,G,H,J,K,M,N)** from P20 to P60. Immunostaining of retinal sections shows Müller and RPE cells (CRALBP marker in green), astrocytes and reactive gliosis (GFAP marker in red), and nuclei (TO PRO marker in blue). General normal retinal structure in C57BL/6J **(A)** and rd10 **(B)** (arrowhead showing the limit between the RNFL + GCL and the IPL) and distribution of Müller cells and astrocytes at P20 **(C–E)**. The ONL and the four outer hyperreflective bands are difficult to recognize from P30 to P40 with OCT **(F,I)**, since the photoreceptors are already degenerating **(G,H,J,K)**. The reactive gliosis is detectable at P30 and is maintained during the disease **(H,K)**. At P60, both the ONL and the outer hyperreflective bands are indistinguishable **(L)** since photoreceptors have degenerated **(M,N)**. Scale bars: **(A–N)** 100 μm.

**FIGURE 2:**
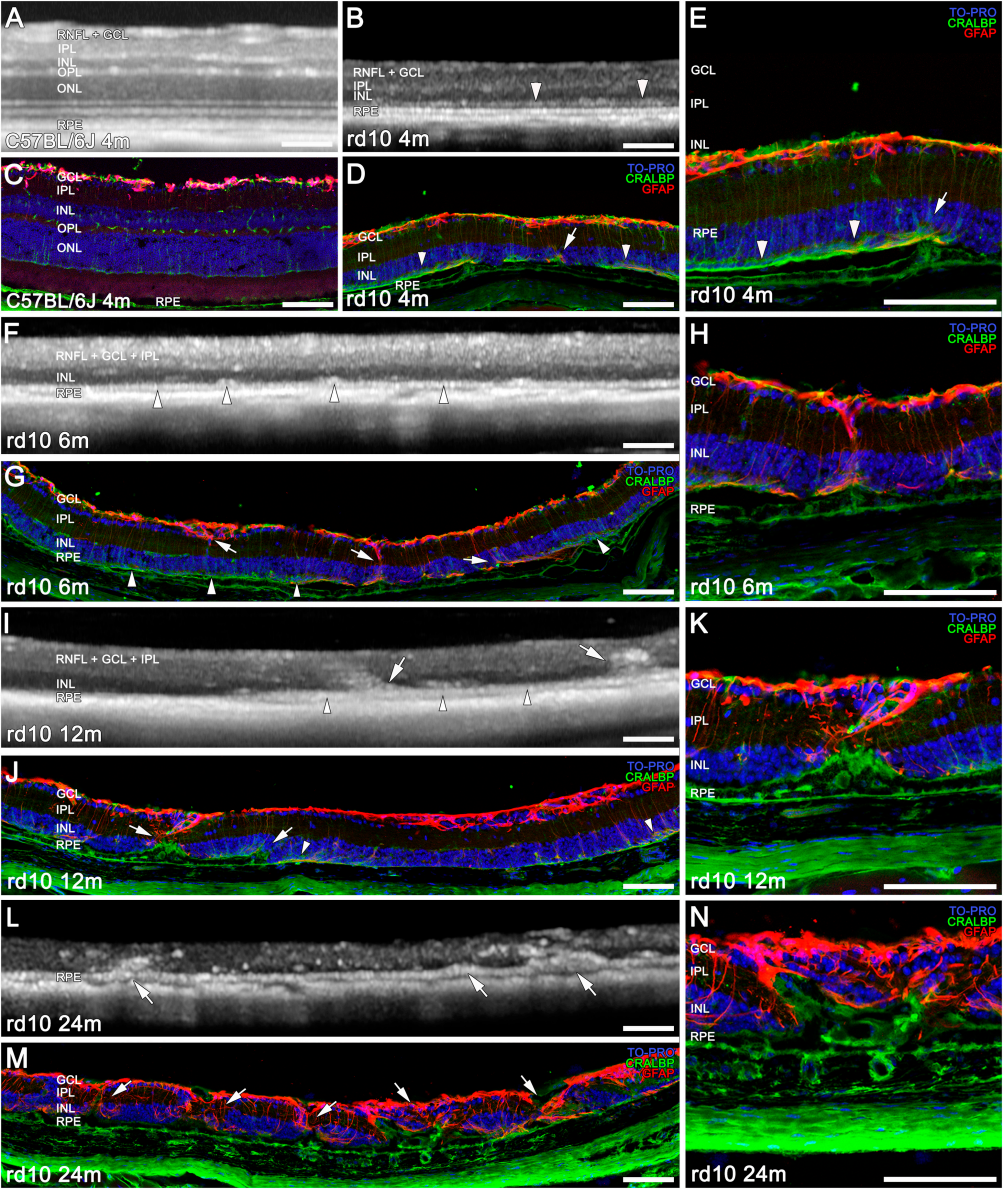
Structural and morphological changes of C57BL/6J and rd10 retinas observed with OCT **(A,B,F,I,L)** and immunohistochemistry **(C–E,G,H,J,K,M,N)** from 4 to 24 m. Immunostaining of retinal sections shows Müller cells and RPE (CRALBP marker in green), astrocytes and reactive gliosis (GFAP marker in red), and nuclei (TO PRO marker in blue). At 4 months, the INL, IPL, and GCL are still generally distinguishable in both animal **(A**vs**B)**; although retinal thickness is already markedly reduced in rd10 compared to C57BL/6J **(C**vs**D,E)**. A band between the RPE and the INL starts forming (**B,D**, arrowheads). Only two bands from the inner retina are detected at 6 and 12 months with OCT **(F,I)** compared to the layers distinguished in IHC (INL, IPL, GCL, RNFL) **(G,H,J,K)**. By 6 months, OCT reveals a distinct hyperreflective outer band above the RPE, and the initial formation of columns (**F**, arrowheads), which corresponds to apical Muller cell processes (**G**, arrowheads). A combination of this band with the remodeling structures is observed at 12 m in the outer retina (**I,J**, arrowheads, arrows). At 24 months, no bands are distinguishable in the OCT **(L)**, and a disorganized retina is observed in IHC **(M,N)**. Remodeling structures can be observed in the rd10 retinas with OCT from 6 months on panels (**F,I,L**, arrows) and in IHC from 4 months on panels (**E** - arrow, **H,K,N**, and in **D,G,J,M**, arrows). Scale bars: **(A–N)** 100 μm.

The OCT was utilized to assess the retinal structure of rd10 mice and correlate these findings with immunohistochemical analyses of age-matched retinas (Figures 1, 2). At P20, OCT images of rd10 retinas revealed a hyperreflective banding pattern similar to that observed in wild-type mice (Figures 1A, B). Within the inner retina, the retinal nerve fiber layer (RNFL) appeared as a distinct hyperreflective band, separable from the ganglion cell layer (GCL) and the inner plexiform layer (IPL) (Figures 1A, B, arrowheads). The inner nuclear layer (INL), and the outer nuclear layer (ONL), were discernible as two hyporeflective bands separated by a hyperreflective band corresponding to the outer plexiform layer (OPL). The outer retina exhibited four distinct hyperreflective bands, indicative of the structural compartments of photoreceptors and the retinal pigment epithelium (RPE) (Figure 1A). However, in the case of the rd10 group, the third and fourth outer bands could not be reliably distinguished (Figure 1B). Histological analysis corroborated these findings, demonstrating well-preserved retinal layering in C57BL/6J and rd10 mice (Figures 1C–E). Immunohistochemical staining with anti-CRALBP antibodies identified Müller cells and the RPE (green), whereas anti-GFAP antibodies labeled astrocytes (red). Nuclei were counterstained with TOPRO (blue) (Figures 1C–E).

By P30, OCT imaging revealed a pronounced reduction in ONL thickness, indicative of substantial photoreceptor loss (Figure 1F). At this stage, histological examination revealed that only two rows of photoreceptor nuclei remained, highlighting a more advanced stage of retinal degeneration compared to P20 (Figures 1E vs 1H). Furthermore, the hyperreflective bands within the outer retina exhibited significant disorganization, consistent with the degeneration of photoreceptors and the progressive disruption of their inner and outer segments (Figure 1F). Immunohistochemical analysis further confirmed these degenerative changes in the ONL, characterized by the depletion of photoreceptor cell bodies and the loss of their inner and outer segments (Figures 1G, H).

From P40 onward, the ONL becomes nearly imperceptible in OCT images (Figure 1I), as only a single row of photoreceptor nuclei remains, as confirmed by TOPRO staining in immunohistochemical analyses (Figures 1J, K). Furthermore, the four outer hyperreflective bands observed in OCT progressively lose their distinction, appearing similar to those seen at P30 (Figures 1F, I). At this stage, the inner retina does not exhibit a significant reduction in thickness.

By P60, the outer hyperreflective bands merge with the remnants of the OPL, rendering them indistinguishable (Figure 1L). The ONL band itself becomes unrecognizable in OCT imaging (Figure 1L), and the immunohistochemical analysis at this time point reveals only a sparse population of remaining photoreceptor nuclei in the ONL (Figures 1M, N).

This degenerative process, which we observed from P30, is accompanied by a progressive increase in GFAP immunoreactivity (red), indicative of reactive gliosis in Müller cells and astrogliosis. This glial response intensifies as retinal degeneration advances, as observed in immunohistochemical images (Figures 1E vs 1H, K, N).

In later stages, the advanced retinal degeneration combined with the low resolution of the images make very difficult to correctly differentiate between all the retinal layers using OCT. Consequently, an immunohistochemical comparison of age-matching retinas is required to elucidate the cellular correspondence with the hypo- and hyperreflective bands (Figure 2). At 4 months, the total retinal thickness is already considerably reduced in rd10 mice compared to C57BL/6J mice, based on both OCT and IHC (Figures 2B, D vs 2A, C). In some regions of the OCT images in rd10 mice, the IPL and INL can be distinguished from the GCL + RNFL (Figures 2A vs 2B). Moreover, at this age, the apical Müller cell processes start forming a band between the RPE and the INL (Figure 2B, arrowheads) that can correspond to the hyperreflective band in the OCT at the same level (Figures 2D, E, arrowheads).

At 6 m, the OCT only allows the distinction of one band above the band corresponding to the RPE (Figure 2F, arrowhead). This band could also be identified by immunohistochemistry as the apical Müller cell processes (Figure 2G, arrowhead). At 12 m, the hyperreflective band distinguished below the INL in the OCT (Figure 2I, arrowheads) can be interpreted as a combination of apical Muller cell processes, RPE, and remodeling structures (Figure 2J, arrowheads and arrows). In both time points (6 and 12 m), the thick hyperreflective band observed by OCT in the innermost retina corresponds to the ganglion cell complex (GCC, which includes the RNFL, GCL, and IPL; Figures 2F, I), as confirmed by the equivalent histological image (Figures 2G, J). Next, a hyporeflective band can be distinguished on top of the pigment epithelium (Figures 2F, I), which would correspond to the INL (Figures 2G, J). A progressive degeneration of the inner retina is observed on OCT between 6 and 12 months (Figures 2F, I). Nevertheless, at these ages, these layers can still be observed separately by IHC (Figures 2E, F, H, I).

Finally, at 24 months of age, there are no bands clearly distinguishable by OCT (Figure 2L), and the histology shows the strong remodeling of the remaining layers of the retina (Figures 2M, N).

### 3.2 Retinal remodeling, characterized by glial and vascular structural alterations, is observed across the entire retina

From 4 m onward, immunohistochemical analysis reveals early signs of inner retinal remodeling (Figure 2D, arrows): CRALBP and GFP immunostaining reveal the initial formation of columns composed of cellular processes extending from the RPE to the GCL. These structures correspond to the invasion of RPE-derived cells (green) into the retina, along with the activation of Müller cells (red) (Figures 2D, G, H, J, K, M, N; arrows). As degeneration advances, these remodeling processes become increasingly prominent. In OCT, a band begins to be discernible at this level (Figure 2B).

From 6 months onward, OCT imaging reveals these previously described structures as hyperreflective columns, which originate from the hyperreflective band corresponding to the RPE and extend into the retina (Figures 2F, I, L; arrows). These invasive processes are detected throughout the retina, extending to the INL and IPL, and ultimately reaching the GCL in all rd10 retinas from 6 m onward (Figures 2I–N, arrows).

**FIGURE 3:**
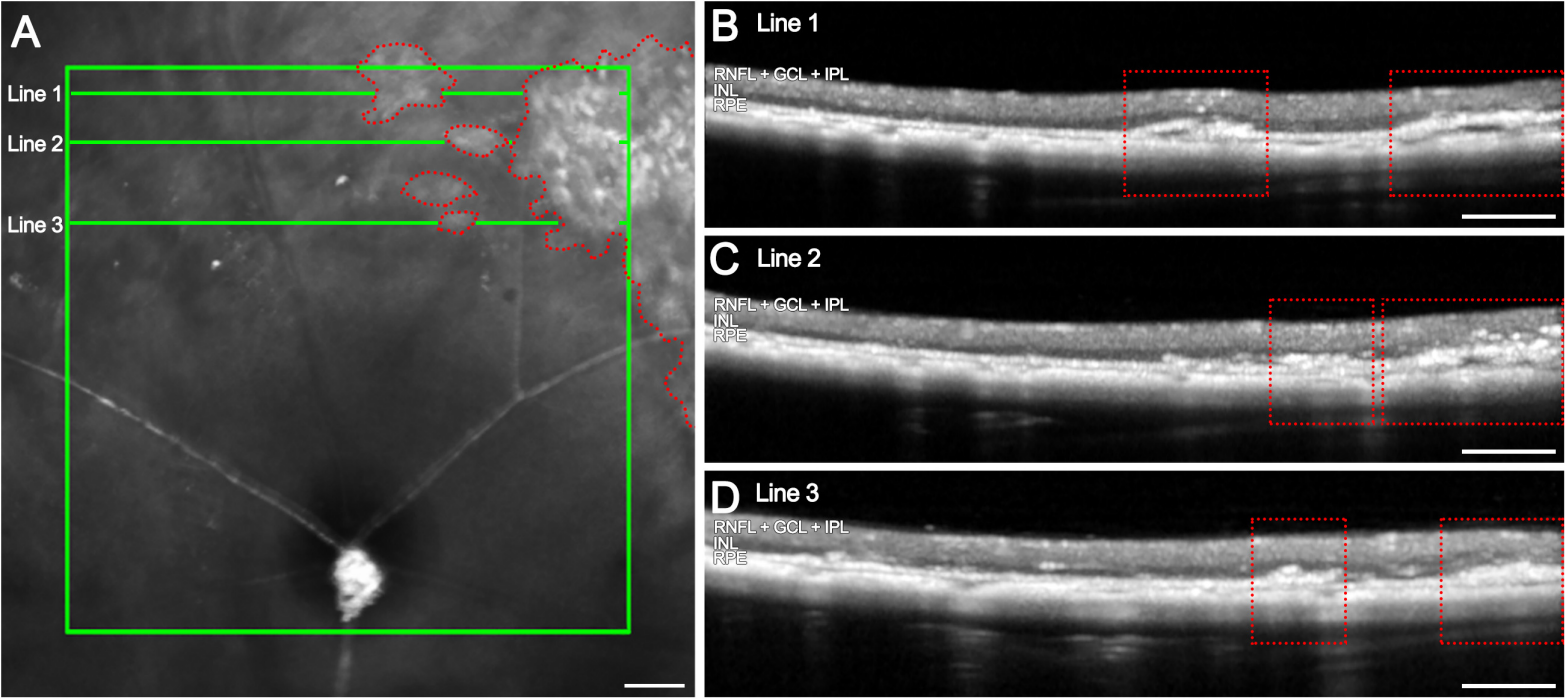
Hyperreflective columns in OCT images of 12-months late-stage rd10 retinas and their correlation with the infrared image. **(A)** Infrared image showing the affected areas in the medial retina (red dotted areas) and the location of horizontal B-scans (line 1, 2 and 3; green). **(B–D)** OCT B-scans at different locations from the optic nerve with numerous columns invading the retina from the RPE up to the IPL (red dotted areas). Scale bars: **(A–D)** 200 μm.

These remodeling structures can be detected using OCT at varying distances from the optic nerve, indicating their widespread distribution across the retinal surface (Figure 3A). In infrared fundus imaging, these columns appear as bright regions, which can serve as reference points for optimizing OCT line acquisition to ensure their detection (Figures 3B–D, red dotted areas). When an OCT scan is acquired at one of these bright regions, the corresponding locations reveal columns extending from the RPE into the retina (Figures 3B–D).

**FIGURE 4:**
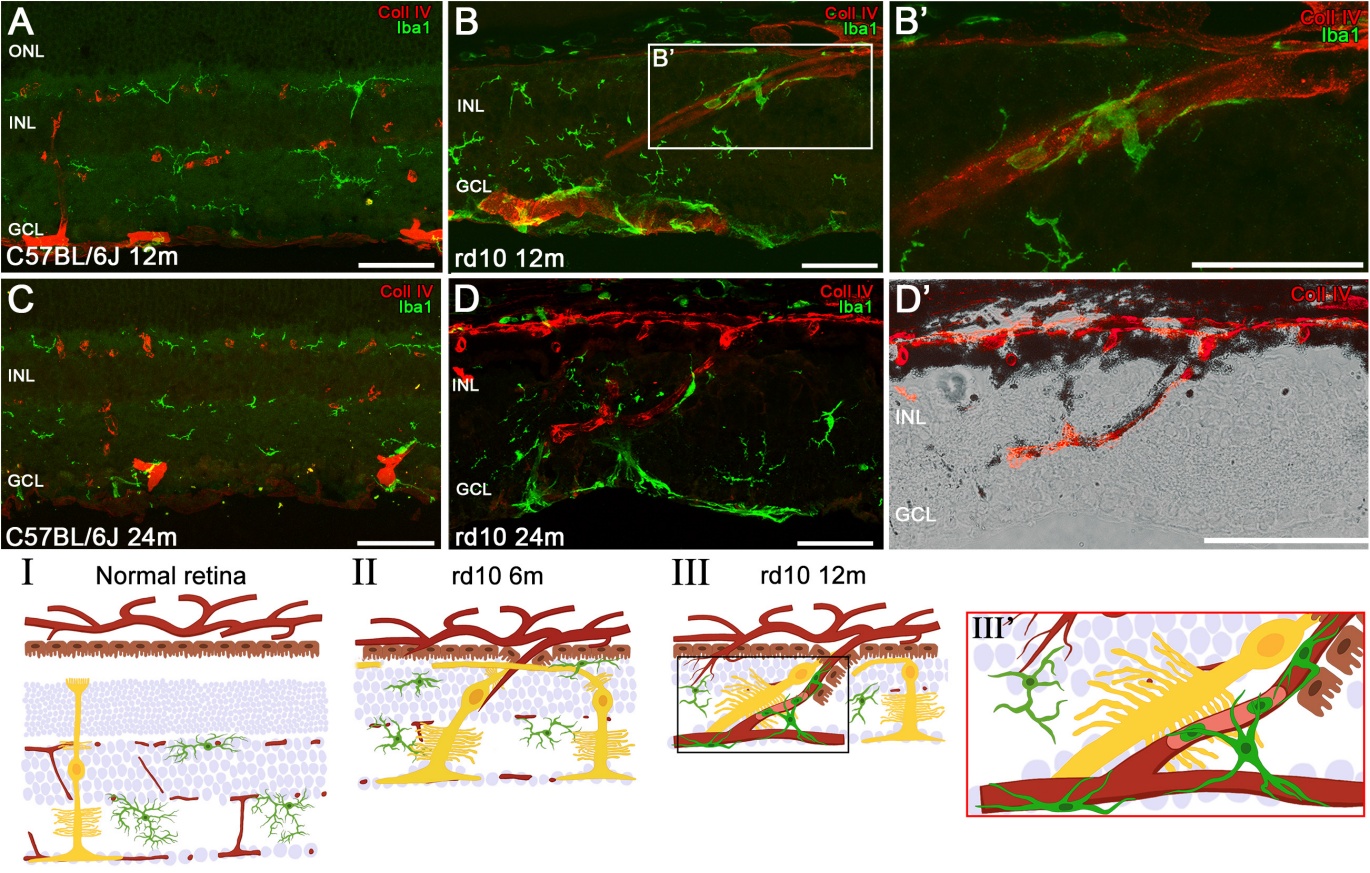
Invasion of the choroidal vessels into the retina and associated microglial changes in late stages of degeneration. Retinal sections of C57BL/6J mice **(A,C)** and rd10 mice **(B,D)** showing the vessels (red, Coll IV) and the microglia (green, iba1) at 12 and 24 months. **(B’)** Magnification of amoeboid microglia in panel **(B)** surrounding the vessel proceeding from the choroid. **(D’)** Detailed image from panel **(D)** highlighting a vessel arising from the choroid in the rd10 mice at 24 m. **(I–III)** Schematic representation of the normal retinal structure **(I)** and the invasion process of choroidal vessels **(II,III)**. Müller cells migrate toward developing vessels at around 6 months **(II)**, followed by the migration of RPE and microglia to these neovessels at 12 months **(III,III’)**. INL, inner nuclear layer; GCL, ganglion cell layer. Scale bars: **(A–D)** 50 μm.

An additional characterization of these structures was performed in C57BL/6J and rd10 retinas at 12 and 24 m using collagen type IV and Iba1 immunostaining (Figure 4). In comparison with the control retinas (Figures 4A, C), these analyses confirm that the columns also contain blood vessels infiltrating the retina from the choroid (Figures 4B, B’). Furthermore, Iba1 staining reveals the presence of microglial cells surrounding the columns, particularly along their inner borders (Figures 4D, D’). These microglial cells exhibit an amoeboid morphology, indicative of an activated state, likely in response to the ongoing retinal degeneration.

A comprehensive schematic representation of the retinal remodeling process is proposed based on immunohistochemical findings, illustrating the involvement of microglia, Müller cells, RPE, and choroidal vessels (Figures 4I–III). In comparison to a normal retina, rd10 retinas at 6 m show that, following the invasion of choroidal vessels into the retina, reactive and hypertrophic Müller cells migrate toward these vessels (Figure 4II). As neovascularization progresses, the RPE extends into the inner retina, accompanied by the recruitment of activated microglia, which accumulate around the invading vessels (Figures 4III, III’). Figure 4II shows the Müller cell processes running between the RPE and the INL.

### 3.3 The rd10 retinal vascular degeneration events detected by OCTA

**FIGURE 5:**
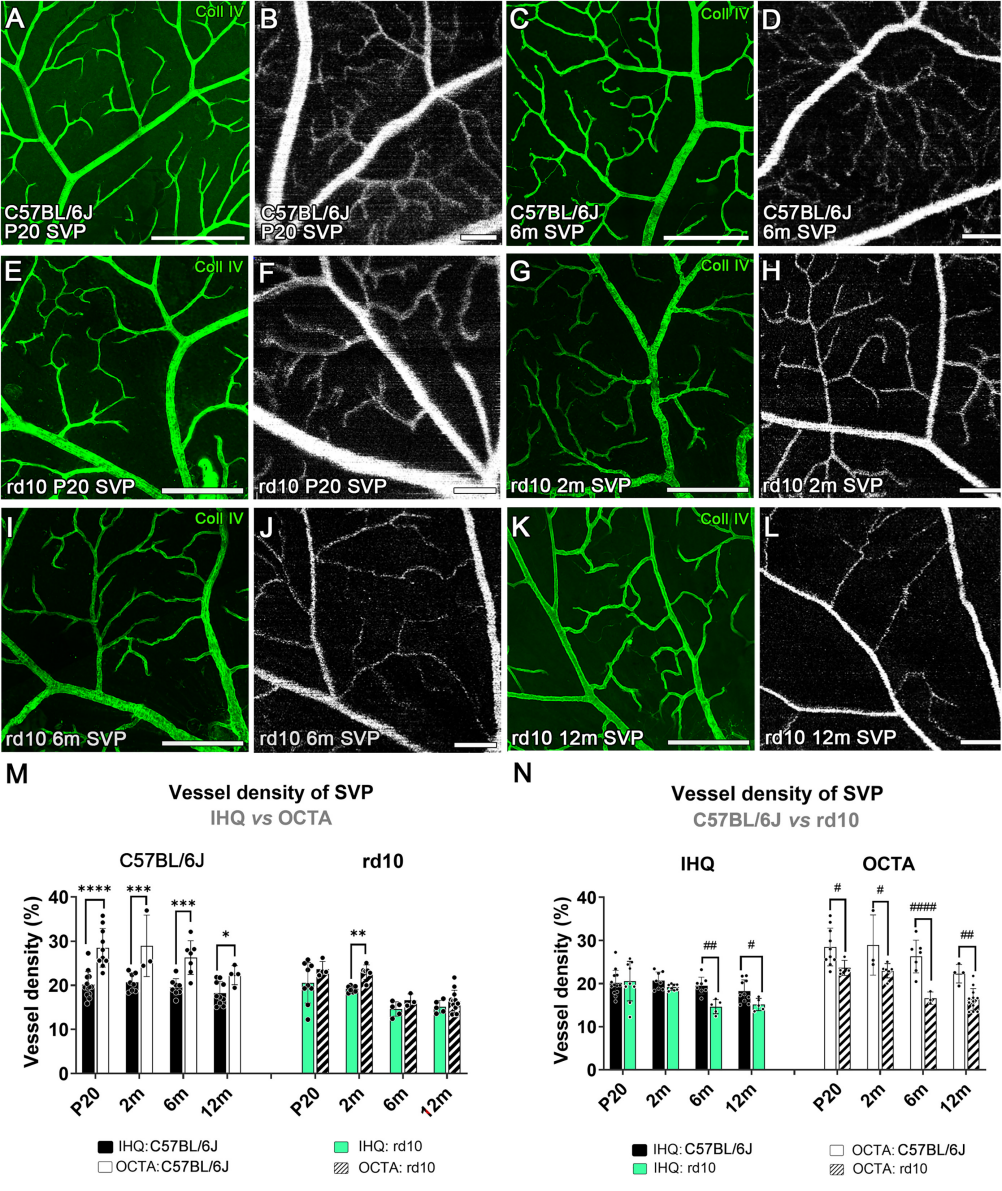
Superficial vascular plexus in confocal immunostained with collagen type IV and OCTA images from C57BL/6J **(A–D)** and rd10 mice **(E-L)** at P20 **(A,B,E,F)**, 2 months **(G,H)**, 6 months **(C,D,I,J)** and 12 months **(K,L)**. **(M)** Vessel density of the superficial vascular plexus analyzed in C57BL/6J (left) and rd10 mice (right), comparing IH with OCTA over time. Results from IHC and OCTA images in the rd10 mice are similar at P20, 6, and 12 months. **(N)** Comparison between C57BL/6J control mice and rd10 mice obtained by IHC and OCTA. Scale bars: **(A–L)** 200 μm. Two-way ANOVA, Bonferroni post-hoc test. *p* < 0.05*; *p* < 0.01**; *p* < 0.001***; *p* < 0.0001****; *p* < 0.05^#^; *p* < 0.01^##^; *p* < 0.0001^####^.

**FIGURE 6:**
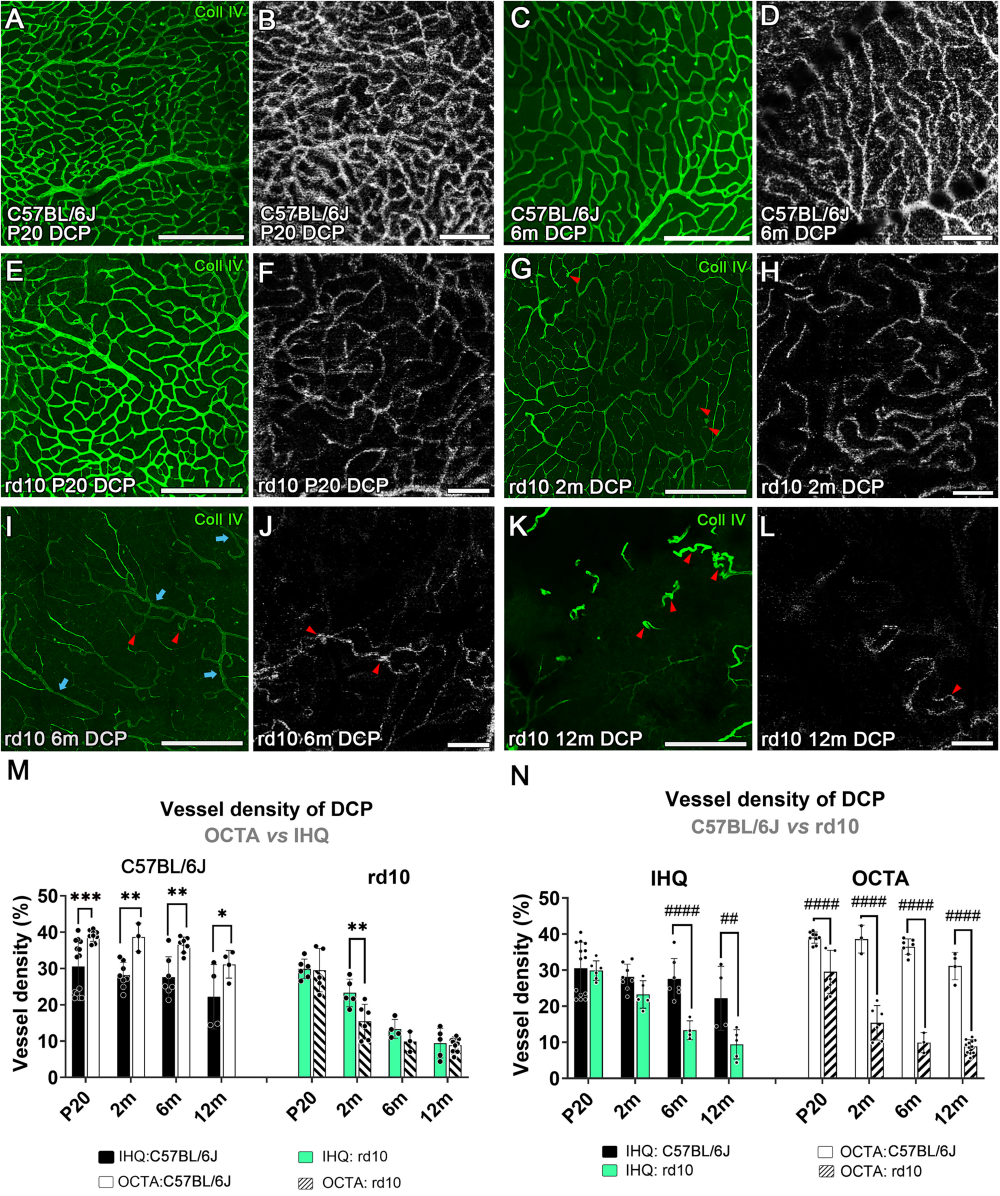
Deep capillary plexus in confocal and OCTA images over time in control and rd10 mice. Comparison between flat-mount retinas immunostained with collagen type IV and OCTA images from C57BL/6J **(A–D)** and rd10 mice **(E–L)** at P20 **(A,B,E,F)**, 2 months **(G,H)**, 6 months **(C,D,I,J)** and 12 months **(K,L)**. Structural and blood flow changes in the vessels are observed at 2 months in rd10 mice **(G,H)** and are exacerbated at 6 months **(I,J)**, compared to control mice **(C,D)**. Red arrowheads, from 2 months onward, indicate capillary bending or capillary loops in the vascular network. Blue arrows highlight the remaining larger-diameter capillaries. **(M)** Vessel density of the deep capillary plexus comparing IHC with OCTA over time in C57BL/6J (left, two-way ANOVA, *p* < 0.0001****) and rd10 mice (right, two-way ANOVA, *p* = 0.01*). Results from IHC and OCTA images in the rd10 mice are similar at P20, 6 and 12 months. **(N)** Comparison between control and rd10 mice results obtained by each method (IHC: left, two-way ANOVA, *p* < 0.0001****; OCTA: right, two-way ANOVA, *p* < 0.0001****). Scale bars: **(A–L)** 200 μm. Two-way ANOVA, Bonferroni post-hoc test. Scale bars: **(A–L)** 200 μm. *p* < 0.05*; *p* < 0.01**; *p* < 0.001***; *p* < 0.01^##^; *p* < 0.0001^####^.

Optical coherence tomography angiography findings were correlated with immunohistochemical analysis using antibodies against collagen type IV at various ages to better understand the progression of vascular remodeling during retinal degeneration (Figures 5, 6). In control animals and at the early stages of degeneration in rd10 retinas (such as P20), the retinal vasculature consists of three distinct vascular plexuses: the superficial vascular plexus (SVP) located in the GCL, the intermediate capillary plexus (ICP) situated in the inner region of the INL, and the deep capillary plexus (DCP) positioned in the outer region of the INL.

#### 3.3.1 Superficial vascular plexus changes in late stages of rd10 retinal degeneration

The vascular density in the SVP of C57BL/6J mice showed no significant differences over time either with IHC (*p* = 0.2) or OCTA (*p* = 0.115, Supplementary Figure 1). However, this density measured using OCTA was significantly higher in all ages compared to that observed with IHC (P20, *p* < 0.0001****; 2 m, *p* = 0.0004***; 6 m, *p* = 0.0002***; 12 m, *p* = 0.042*; Figure 5M-left, and Table 1).

**TABLE 1:** Vascular density with immunohistochemistry and OCT-A techniques.

Vascular plexus	Group	Age	Vessel density,% (*n*)		
			Immunohistochemistry	OCT-A	*P*-value
Superficial vascular plexus (SVP)	C57BL/6J	P20	20 ± 3 (13)	29 ± 4 (10)	*P* < 0.0001****
		2 months	20 ± 2 (8)	29 ± 7 (3)	*P* = 0.0004***
		6 months	20 ± 2 (8)	26 ± 4 (7)	*P* = 0.0002***
		12 months	18 ± 3 (10)	22 ± 2 (4)	*P* = 0.042*
	rd10	P20	20 ± 5 (9)	24 ± 2 (4)	*P* = 0.051
		2 months	19.2 ± 0.7 (8)	23 ± 2 (6)	*P* = 0.009**
		6 months	15 ± 2 (5)	16 ± 1 (3)	*P* = 0.301
		12 months	15 ± 1 (5)	16 ± 3 (12)	*P* = 0.389
Deep capillary plexus (DCP)	C57BL/6J	P20	30 ± 7 (14)	39 ± 2 (9)	*P* = 0.0004***
		2 months	28 ± 4 (8)	39 ± 4 (3)	*P* = 0.005**
		6 months	28 ± 6 (7)	36 ± 2 (7)	*P* = 0.003**
		12 months	22 ± 9 (4)	31 ± 4 (4)	*P* = 0.02*
	rd10	P20	30 ± 3 (6)	30 ± 6 (5)	*P* = 0.875
		2 months	23 ± 4 (5)	15 ± 5 (6)	*P* = 0.001**
		6 months	13 ± 3 (4)	10 ± 3 (3)	*P* = 0.216
		12 months	9 ± 4 (5)	9 ± 2 (12)	*P* = 0.768

Bonferroni’s multiple comparisons from Two-Way ANOVA. *n*: sample size. *p* < 0.05*; *p* < 0.01**; *p* < 0.001***; *p* < 0.0001****.

In rd10 mice, the decrease of the vessel density was significant over time when analyzed through IHC (*p* = 0.002**) or OCTA (*p* < 0.0001****, Supplementary Figure 1). A peak in SVP degeneration over time was observed between 2 and 6 months, which was statistically significant by OCTA (*p* = 0.003**) but not by IHC (*p* = 0.066) (Supplementary Figure 1). No changes in density were observed between 6 and 12 months using any of the techniques in rd10 mice (Supplementary Figure 1). When comparing the two techniques, measurements of vascular density in rd10 mice obtained by OCTA and IHC were comparable over time, except at 2 months (*p* = 0.009**; Figure 5M-right, and Table 1).

Comparison between rd10 and control mice revealed significant differences in the SVP (Figure 5N). Immunohistochemistry analysis revealed that vessel density of rd10 mice was significantly lower than in control mice (*p* = 0.02*), specifically at 6 months (*p* = 0.002**) and at 12 months (*p* = 0.039*) (Figure 5N-left). In contrast, OCTA imaging detected differences in vessel density between rd10 and control mice much earlier, starting from P20 (*p* < 0.0001****; Figure 5N-right).

#### 3.3.2 Deep capillary plexus degenerates in both animal models

Progression changes over time were also analyzed in the DCP for each group (Supplementary Figure 2). In C57BL/6J mice, the DCP revealed a slight reduction in vessel density with age. These changes were not statistically significant when assessed by IHC (*p* = 0.169), but a progressive reduction in vessel density was detected using OCTA from 2 to 12 months (*p* = 0.00048***) (Supplementary Figure 2). Similarly to the observed for SVP, in the control mice, the OCTA values were significantly higher than those for IHC in all ages (*p* < 0.0001****, Figure 6M-left, and Table 1).

Progressive degeneration in DCP was observed in rd10 mice over time using both IHQ (*p* < 0.0001****) and OCTA (*p* < 0.0001****, Supplementary Figure 2). The most significant peak of vascular degeneration was observed between 2 and 6 months using IHQ (*p* = 0.003**) while OCTA imaging showed the peak earlier, between P20 and 2 months (*p* < 0.0001****, Supplementary Figure 2). Qualitative observations revealed that the earliest signs of vascular degeneration detected by IHQ from 2 months included specific structural changes such as vessel thinning with acellular capillaries, capillary bending, and capillary loops (Figures 6G, I, K, red arrowheads). By 6 months, only a few larger-diameter capillaries remained (Figure 6I, blue arrows). On the contrary, the vascular signs found in the OCTA images differed from the IHQ. The earliest sign of vascular degeneration found by OCTA was the loss of perfusion in some areas at P20 (Figure 6F). Then, only vessels with sustained blood flow were detectable at both 2 and 6 months, likely corresponding to those with larger diameters (Figures 6H, J). The thin-diameter capillaries identified through immunohistochemistry were not visible in OCTA, possibly due to a lack of active blood circulation within these structures (Figures 6I vs 6J). At 12 months, marked degeneration of the DCP was observed with both techniques (IHC and OCT) revealing the presence of abnormal, isolated vessel tangles with dead-ends (Figures 6K, L, arrowheads). Furthermore, specific differences in the vascular density between methods for the rd10 group are also shown in Figure 6M-right. No differences were observed between IHC and OCT measurements at any time point, except at 2 months (Figure 6M-right, and Table 1).

Finally, differences in the vessel density of the DCP between control and rd10 for each method were assessed (Figure 6N). The structural differences by the IHC analysis between control and rd10 mice were detected from 6 months (*p* = 0.0002***, Figure 6N-left) onward. However, the vessel density quantified with OCTA exhibited differences between control and rd10 mice from P20 up to 12 months (*p* < 0.0001****, Figure 6N-right),

#### 3.3.3 Global changes in the whole-mount retinas

**FIGURE 7:**
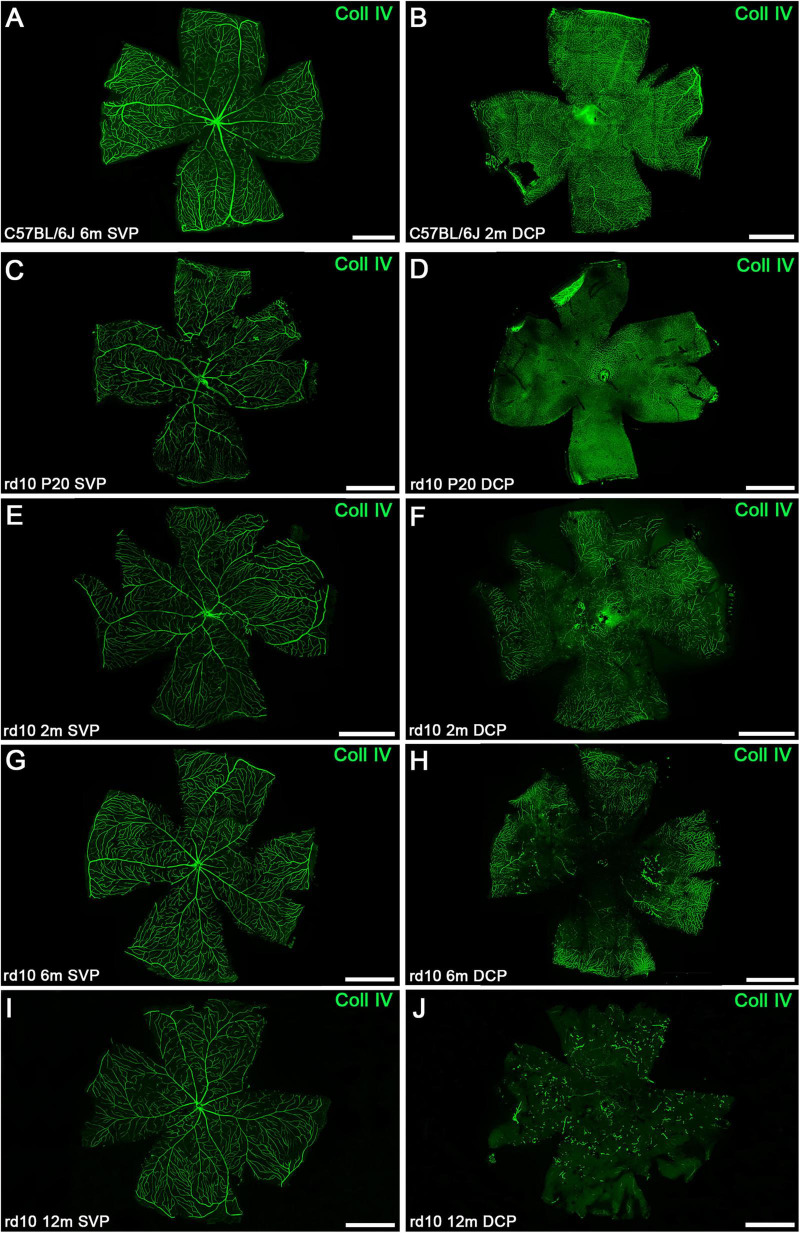
Representative whole-mount retinas from C57BL/6J and rd10 mice at the SVP and DCP over time immunostained with collagen type IV. **(A,B)** Representative images of the SVP **(A)** and DCP **(B)** from control retinas at 6 and 2 months, respectively. Representative images of the retinal SVP **(C,E,G,I)** and DCP **(D,F,H,J)** from rd10 mice during the degenerative process. The structural degeneration of DCP appears at 2 months **(F**vs**B)**. **(H)** The loss of central retinal vasculature is more pronounced by 6 months. **(J)** At 12 months, abnormal dead-end isolated vessel tangles were present in the rd10 mice. SVP, superficial vascular plexus; DCP, deep capillary plexus; Coll IV, collagen type IV. Scale bars: **(A–J)** 1 mm.

Figure 7 exhibits representative IHC images of whole-mount retinas over time, showing the SVP and DCP. As supported by the quantitative analysis, changes in the SVP of rd10 mice over time were not clearly distinguished. Nevertheless, whole-mount retina analysis further confirmed DCP degeneration in rd10 mice (Figures 7D, F, H, J). Vessel loss or thinning can be distinguished at 2 months (Figure 7F), with degeneration becoming more evident by 6 months, particularly in the central retina. Notably, vascular degeneration in the DCP initially appears in the central region at 6 months and progressively extends toward the periphery by 12 months (Figure 7H). Morphological changes in both plexuses can be visualized across the entire retina at advanced ages (Figures 7I, J).

## 4 Discussion

The advent of novel *in vivo* imaging technologies has revolutionized the assessment of retinal and optic nerve pathologies in clinical settings. These advanced tools, widely used in clinical practice, have also proven highly effective in preclinical research. Retinal imaging OCT now enables high-resolution visualization of retinal structures in small animal models, facilitating the investigation of the progression of retinal degenerative diseases and the evaluation of therapeutic interventions (Cuenca et al., 2014a; Berger et al., 2014). These techniques offer significant advantages, including the reduction of the number of animals required for studies and the ability to track retinal degeneration over time.

Indeed, our study demonstrates that OCT and OCTA are valuable tools for monitoring long-term retinal and vascular changes in the rd10 model up to 12 months of age, when compared with traditional IHC techniques. Previous studies have used OCT and OCTA to investigate rd10 mice retinal degeneration, but primarily at relatively early stages of the disease (Hasegawa et al., 2016; Kim et al., 2018) or up to 5 months (Pennesi et al., 2012). For instance, Kim et al. (2018) reported vascular alterations up to P28, and Hasegawa et al. (2016) studied structural retinal changes up P56. In addition, OCTA and OCT have been applied to study vascular alterations focusing on hyaloid vessel regression (Kim et al., 2019, 2020) or the optoretinography of photoreceptor dysfunction in the rd10 mice. Additionally, we have described the remodeling structures associated with disease progression in the rd10 model using both OCT and IHC techniques. These remodeling structures, which become apparent from 6 months onward, consist of choroidal vessels that invade the retina and are surrounded by Müller cells, microglia, and RPE.

Our findings in rd10 mice reveal a strong correlation between anatomical changes observed via OCT and IHC, particularly in early disease stages when retinal layers are clearly distinguishable. Both techniques detected an initial reduction in retinal thickness due to photoreceptor loss and ONL thinning. After photoreceptor degeneration (around P30), the INL and IPL remained identifiable by OCT. At later stages, the INL became disrupted while the IPL appeared preserved, consistent with observations in other models (Cuenca et al., 2014b). However, OCT lacks the resolution to visualize cellular or synaptic changes or to detect advanced disease modifications, clearly evident in IHC images.

Although the ONL and the outer bands degenerate rapidly in this model, making difficult to distinguish by OCT at advanced ages, the technique remains useful for detecting structural changes associated with retinal remodeling. Here, we evidence the existence of columns that disrupt the remaining layers in the rd10 retina, reflecting an invasion of blood vessels surrounded by RPE, associated with activated glial cells such as Müller cells and microglia. This coincides with the results in other models of RP, as the P23H or the RCS rats (Pinilla et al., 2016; Jones and Marc, 2005; Marc and Jones, 2003; Marc et al., 2003), where similar structures can be found. Moreover, the involvement of glial cells in the degeneration process observed in RP models has been widely described (Noailles et al., 2014, 2016; Zhao et al., 2015; Roche et al., 2016a; Fernández-Sánchez et al., 2015). Due to their feature of becoming reactive in response to tissue perturbation, glial cells are expected to occupy the area where the retinal disruption occurs. It is also worth mentioning that microglial cells may contribute to the disrupting columns hyperreflectivity, since other authors have hypothesized that these cells might be responsible of hyperreflective structures that appear in age-related macular degeneration (Curcio et al., 2017). We also characterized a newly observed hyperreflective band, clearly visible from 4 months onward, located between the RPE and the INL. This band is formed by the processes of Müller cells, which align parallel to the RPE following the degeneration of photoreceptors. The outer part of Müller cells is located between photoreceptor nuclei, so the loss of photoreceptors consequently leads to the displacement of Müller cells on the inner retina. Therefore, the presence of these displaced structures reflects substantial photoreceptor loss, and based on our results, their detection and identification may provide valuable information about the state of the retina and glial cells.

A well-established observation is that the retina undergoes significant transformation and remodeling following photoreceptor loss. In our long-term rd10 mouse model of retinal degeneration (up to 24 months of age), we observed Müller cell hypertrophy, migration of surviving neurons, and RPE migration traversing the retina, accompanied by vascular remodeling. In OCT imaging, columns of hyperreflectivity were evident during retinal degeneration. Through immunohistochemical correlation, we determined that these hyperreflective structures represent remodeling complexes composed of blood vessels, Müller cells, RPE, and microglia.

Another key structure affected in the advanced stages of retinal degeneration is the retinal vasculature. Under normal physiological conditions, photoreceptors have a high oxygen demand and contain numerous large mitochondria, with the highest concentration located in the ellipsoid region. In RP, as photoreceptors degenerate, the substantial oxygen supply to the retina is no longer efficiently utilized, creating a hyperoxic environment. In response to this oxidative stress, retinal blood vessels undergo retraction, likely as a protective mechanism to limit excessive oxygen exposure and prevent oxidative damage to the remaining retinal tissue (Cuenca et al., 2014a; Fernández-Sánchez et al., 2018).

In this work, we demonstrate for the first time that the vessel density measurements obtained via OCTA and immunohistochemistry provide comparable long-term results in the rd10 mice in both the SVP and the DCP across most ages tested. This correlation occurs despite the IHC assesses the structural component of the vessels, while OCTA imaging results from the blood flow. Therefore, the correlation between these techniques suggests that the structural degeneration of vessels coincides with the reduction in blood flow in rd10 mice. These findings advocate for the use of non-invasive techniques in vessel studies in the rd10 animal model, given the challenges and limitations associated with working with flat-mounted retinas. There is a previous study that employs the OCTA technique to analyze the vascular integrity of rd10 retinas, but it focuses on the early stages of the disease, up to P28 (Kim et al., 2018). Our OCTA results in the deep plexus at P20 confirm these previous results.

According to our results, the 2-months time point appears to mark a change in both the SVP and DCP, as it is the only time point where differences between the two techniques’ results were observed (Figures 5M, 6M). This may suggest that, at this stage, the DCP is experiencing a greater reduction in blood flow than a structural loss. In contrast, the SVP shows more pronounced structural changes in the vessels. Specifically, the decrease in blood flow observed with OCTA at 2 months in the DCP is proposed to be related to a reduced metabolic load and a hypoxic state in the retina, both resulting from photoreceptor loss (Grunwald et al., 1996; Verbakel et al., 2018). This could explain the blood-retina barrier failure that occurs in the rd10 mice (Kim et al., 2018; Ivanova et al., 2019). Indeed, described that a hypoxic environment could attenuate vascular degeneration in a model of retinitis pigmentosa, suggesting that retinal vessels can respond to metabolic demands beyond simply undergoing atrophy or growth. In the SVP, greater degeneration of the vessel structure may be linked to thickening of the extracellular matrix around leaky vessels, which leads to narrowing of the vascular lumen (Li et al., 1995). The degeneration in this model is similar to that occurring in other RP models, like the P23H rat, which presents a decrease of the capillary density and the number of capillary loops in the DCP at 4 months and ending with a total loss of the plexus at 16 months (Fernández-Sánchez et al., 2018). Moreover, the abnormalities that appear at 12 months as isolated vessel tangles in rd10 mice were already seen at 13 months in previous studies using immunohistochemistry (Ivanova et al., 2019).

On the other hand, few studies have analyzed the SVP in RP animal models (Kim et al., 2018; Zhang et al., 2023; Wang et al., 2024). In our study, we observed a reduction in superficial plexus density at 6 months in rd10 mice compared to controls, using both imaging techniques. This decline in the vascular network may be followed by the inner retinal degeneration typically observed at later disease stages (Cuenca et al., 2014a; Marc et al., 2003). These findings differ from those reported by Kim et al. (2018), who observed changes in this plexus compared with C57BL/6J as early as P21. Such differences may be explained, at least in part, by variations in experimental design and methodological parameters (Kim et al., 2018) including light intensity conditions, which were not specified in their work but are known to significantly influence the course of degeneration (Kutsyr et al., 2020). Importantly, both studies highlight that vascular alterations in the SVP occur in association with neurodegenerative changes, although the precise onset may vary depending on the experimental conditions. Although SVP degeneration has been reported in RP patients, the clinical stage of the disease is not always clearly defined, which limits the ability to draw direct comparisons with animal models (Ling et al., 2019; Sugahara et al., 2017). However, previous studies seem to find SVP degeneration at mid- or late stages of RP. Koyanagi et al. (2018) described degenerative changes in the SVP of patients with markedly reduced or non-recordable ERG responses, which would typically indicate more advanced stages of the disease. Toto et al. (2016) observed similar results of SVP degeneration in patients with mid- to late-stage RP. In any case, the DCP exhibits more pronounced degeneration than the SVP, indicating a greater vulnerability of the deeper vascular network during retinal degeneration.

Regarding the vascular network in the C57BL/6J mice, the measurements of OCTA vascular density showed higher values for both the superficial and deep plexuses compared to those obtained through immunohistochemistry from P20 to 6 months. Similar differences have also been detected in the vessel diameter by other authors (Duggan et al., 2020; Yao et al., 2021). These results may stem from the overestimation of density due to the oversampling rate and/or image artifacts associated with lateral resolution limitations (Yao et al., 2021). Based on our findings in rd10 mice, where such differences between OCTA and immunohistochemistry results are absent in most ages, it seems that these limitations do not significantly impact when a degenerative process is assessed.

As can be seen in this work, the OCTA enables the assessment of the specific plexuses where degeneration occurs in the rd10 mice. However, this technique cannot yet replace immunohistochemistry since the details obtained by this methodology are far from those achieved by OCT images. Both techniques are complementary for the study of retinal degeneration, contributing with *in vivo* and post-mortem results. Moreover, considering the difficulty of separating the plexuses in a degenerative context, it is important to achieve the correct OCTA segmentation.

## 5 Conclusion

Optical coherence tomography and OCTA, in combination with immunohistochemistry, allow the long-term evaluation of retinal and vascular degeneration in the rd10. In this context, the *in vivo* monitoring of the retinal remodeling using OCT and OCTA could provide a novel approach to evaluate retinal pathology. Moreover, remodeling-associated structures should be considered when interpreting images from these techniques in both clinical practice and basic science. Last, the knowledge of the integrity of cellular organization at advanced stages of degeneration is crucial for the effectiveness of applied therapies, as it helps identify which cellular circuitries remain viable targets.

## Data Availability

The raw data supporting the conclusions of this article will be made available by the authors, without undue reservation.
