# Supplementary material for: Vascular degeneration and retinal remodeling in rd10 mice: correlating OCT, OCTA, and histological findings
Source: Front Neuroanat. 2025 Oct 21;19:1683877. doi: 10.3389/fnana.2025.1683877 (PMC12582963; doi:10.3389/fnana.2025.1683877)
Supplement: Supplementary file 1 [file Data_Sheet_1.docx]

**SUPPLEMENTARY MATERIAL**


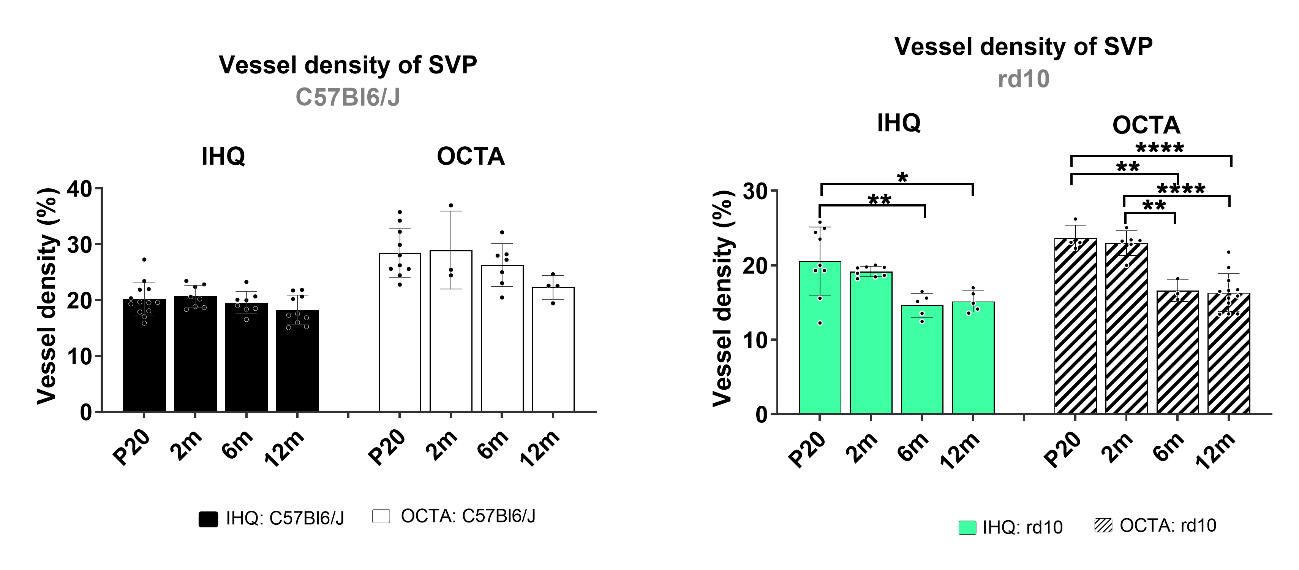


**Figure 1S. Vascular density progression changes in the superficial vascular plexus of control C57BL/6J (a) and rd10 mice (b) using immunohistochemistry and OCTA.** (a) No significant age-related changes were observed in C57BL/6J mice, either by IHQ (*p* = 0.2, left) or OCTA (*p* = 0.115, right). **(b)** In rd10 mice, progressive vascular degeneration was detected over time using both IHQ (left, *p* = 0.002) and OCTA (right, *p* < 0.0001). Results from two-way ANOVA (a, b).


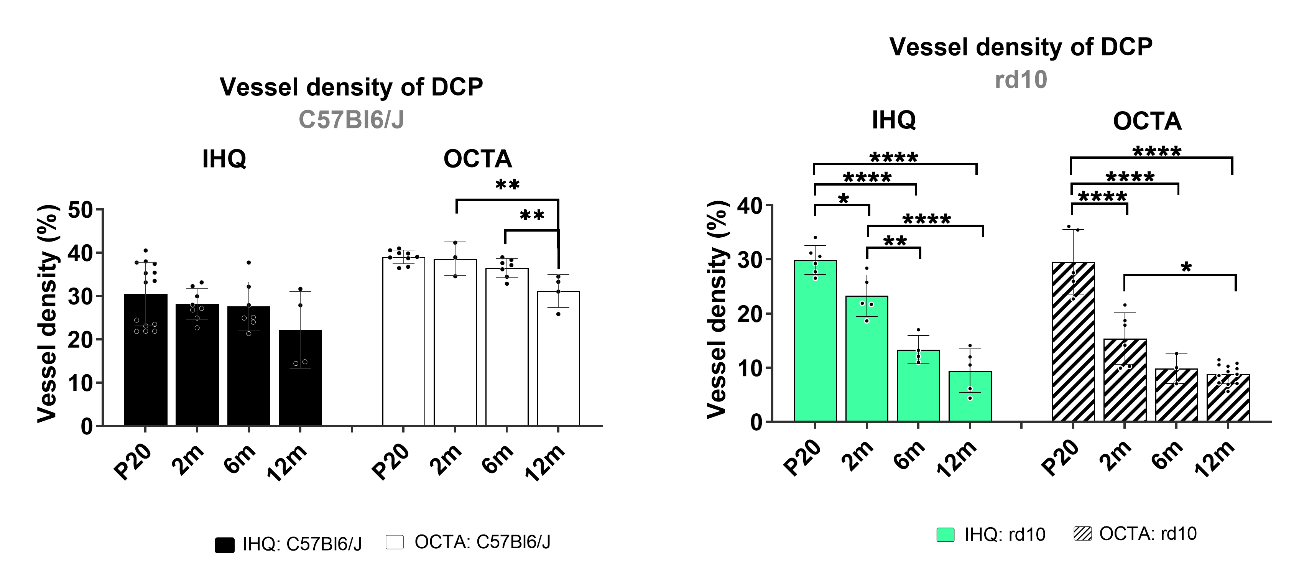


**Figure 2S. Vascular density progression changes in the deep capillary plexus of control C57BL/6J (a) and rd10 mice (b) using immunohistochemistry and OCTA**. (a) No significant age-related changes were observed in C57BL/6J mice by IHQ (*p* = 0.169, left), but significant changes were detected with OCTA (*p* < 0.001, right). **(b)** In rd10 mice, progressive vascular degeneration was detected over time using both IHQ (left, *p* < 0.0001) and OCTA (right, *p* < 0.0001). Results from two-way ANOVA (a, b).
